# Maintaining ecosystem resilience: functional responses of tree cavity nesters to logging in temperate forests of the Americas

**DOI:** 10.1038/s41598-017-04733-2

**Published:** 2017-06-30

**Authors:** José Tomás Ibarra, Michaela Martin, Kristina L. Cockle, Kathy Martin

**Affiliations:** 1 Department of Forest and Conservation Sciences, University of British Columbia, Vancouver, British Columbia Canada; 2 Centre for Local Development, Education and Interculturality (CEDEL), Villarrica Campus, Pontificia Universidad Católica de Chile, La Araucanía Region, Chile; 3 Fauna Australis Wildlife Laboratory, Department of Ecosystems and Environment, School of Agriculture and Forest Sciences, Pontificia Universidad Católica de Chile, Santiago, Chile; 4 Instituto de Bio y Geociencias del NOA (IBIGEO-CONICET-UNSa), Salta, Argentina; 5 Environment & Climate Change Canada, Pacific Wildlife Research Centre, Delta, British Columbia Canada

## Abstract

Logging often reduces taxonomic diversity in forest communities, but little is known about how this biodiversity loss affects the resilience of ecosystem functions. We examined how partial logging and clearcutting of temperate forests influenced functional diversity of birds that nest in tree cavities. We used point-counts in a before-after-control-impact design to examine the effects of logging on the value, range, and density of functional traits in bird communities in Canada (21 species) and Chile (16 species). Clearcutting, but not partial logging, reduced diversity in both systems. The effect was much more pronounced in Chile, where logging operations removed critical nesting resources (large decaying trees), than in Canada, where decaying aspen *Populus tremuloides* were retained on site. In Chile, logging was accompanied by declines in species richness, functional richness (amount of functional niche occupied by species), community-weighted body mass (average mass, weighted by species densities), and functional divergence (degree of maximization of divergence in occupied functional niche). In Canada, clearcutting did not affect species richness but nevertheless reduced functional richness and community-weighted body mass. Although some cavity-nesting birds can persist under intensive logging operations, their ecosystem functions may be severely compromised unless future nest trees can be retained on logged sites.

## Introduction

Resilience is the capacity of an ecosystem to adaptively overcome disturbance while maintaining its essential structures and functions^1, 2^. Anthropogenic disturbances, however, are pushing some ecosystems beyond thresholds of resilience, with a resultant global wave of biodiversity loss^3, 4^. To understand the potentially large ecosystem consequences of these disturbances, ecologists have extended the conventional assessments of how disturbances affect taxonomic diversity (e.g., species richness), to how they affect ecosystem function^3, 5^. Functional diversity, defined as the value, range, and density of functional traits (behavioral, morphological, physiological) in ecological communities, mechanistically links biodiversity and rates of ecosystem functioning^6^. Understanding how anthropogenic disturbances influence both taxonomic and functional diversity can assist policy makers in the design of strategies for the maintenance of ecosystem resilience^7, 8^.

Forest ecosystems are globally exceptional for their contributions to conserving biodiversity and maintaining resilience against environmental instability, but their global surface continues to shrink as a result of logging and conversion to other land uses^9^. Among the biomes most affected by industrial logging are the temperate forests of North and South America, where old-growth forests have been reduced to only 3% of their original extent^9–12^. Globally, the most extensive areas of temperate rain forests occur along the northern Pacific coast of North America (46–61°N), including a broad band reaching part of interior British Columbia, and across a similar latitudinal range (35–56°S) in southern Chile^13, 14^. These forests include some of the longest-lived and massive tree species in the world and share a history of rapid climatic change^13, 15^. There is growing concern that rates of temperate forest loss are negatively affecting forest-dwelling biodiversity in both southern and northern hemispheres^10, 16, 17^.

Logging treatments vary in their impact on forest wildlife and some may even improve conditions for tree-dependent biodiversity when important habitat structures are intentionally retained over the long term^18–20^. Standing dead and decaying trees are critical stand-level habitat structures that provide sites for the reproduction and shelter of more than 1,000 cavity-nesting bird species globally^21, 22^. Tree cavity-using vertebrates participate in a diversity of ecosystem processes, including cavity creation, pollination, mechanical damage to trees (facilitating colonization by fungi), dispersal of seeds and fungi, and control of invertebrate populations^23, 24^. To conserve cavity-nesting birds and their ecosystem functions across temperate forests of North and South America, it is important to investigate how the functional diversity of these communities responds to different logging treatments^25, 26^.

**Figure 1:**
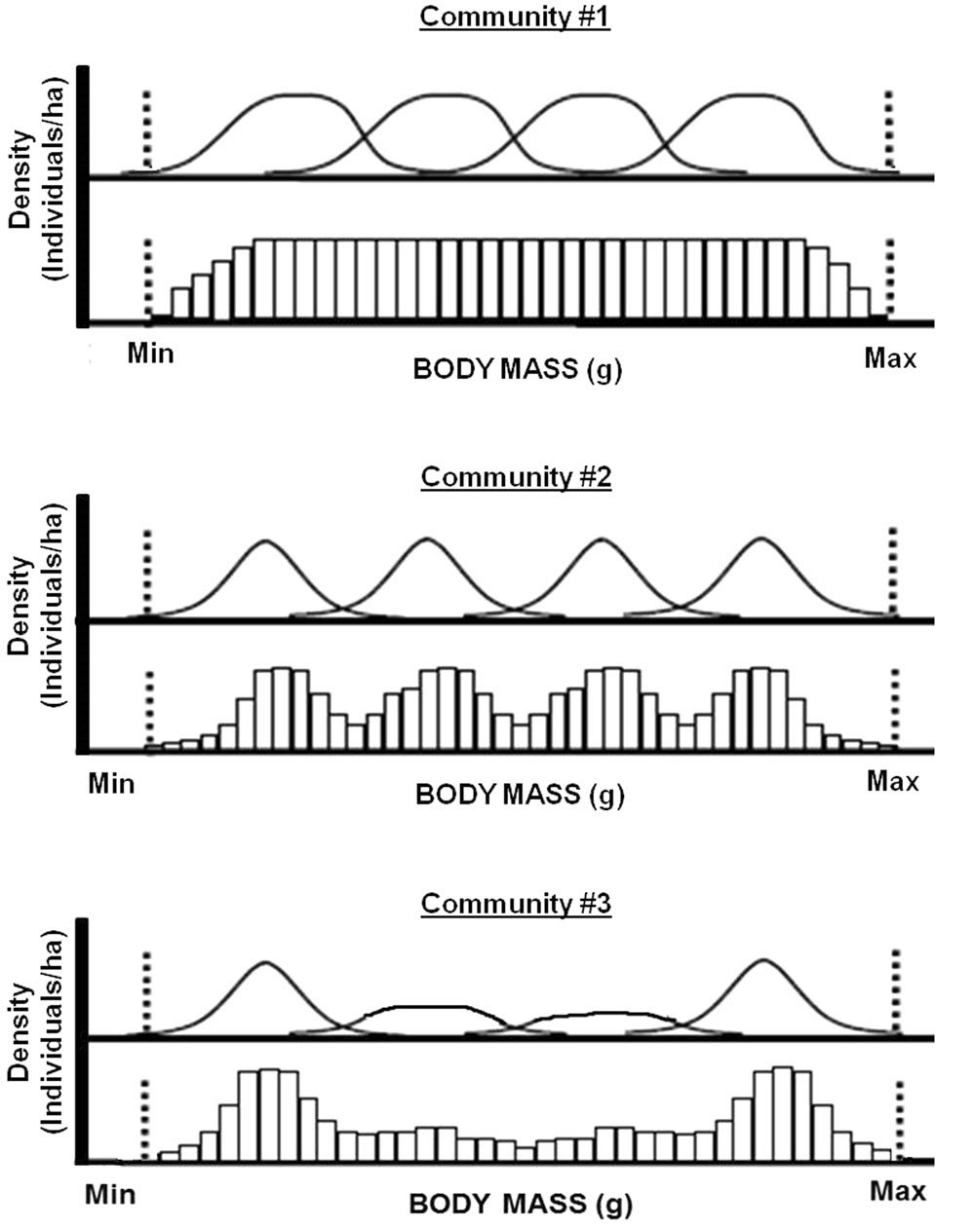
Three hypothetical communities that differ in functional evenness (FEve) and functional divergence (FDiv), but not functional richness (FRic) for a single functional trait (body mass). The vertical dotted lines indicate the boundaries of the niche volume filled by all species together. The y-axes represent density of individuals. The solid curved lines depict the distribution of the density of individuals in the functional niche volume. The histograms show the summed density of individuals across species occurring in the functional niche volume (i.e. equal-width sections of the functional trait range). Community #1 has higher FEve than communities #2 and #3 (densities are more evenly distributed within the filled functional niche volume [in this case 1-dimensional]). Community #3 has a higher FDiv than communities #2 and #3 (species with the highest density of individuals occur at the extremities of the filled functional niche; Modi﻿fied from 31).

Using functional diversity parameters, including functional richness (FRic), functional evenness (FEve), functional divergence (FDiv), and community-weighted mean^27^ (CWM; definitions in Table 1; Fig. 1), researchers have revealed the major role of habitat filtering (constraints selecting species according to their functional traits) as a driver of community assembly^27, 28^. Forest logging may act as an “anthropogenic habitat filter”, removing certain functional traits in a community^5, 29^. For example, disturbances may decrease the functional differentiation among co-occurring species, reducing functional richness^30, 31^. However, systems with relatively more species generally show higher functional redundancy (organisms resembling each other in their functional traits) such that the resilience of functional diversity to disturbance is predicted to be higher in species-rich than in species-poor systems^32^. Functional evenness and functional divergence in fish and avian communities have either decreased or shown no shifts in response to anthropogenic habitat filtering^33, 34^. Community-weighted mean (CWM) of trait values has been linked to the maintenance of ecosystem functions such as nutrient recycling by carabid beetles, pollination by bees, and pest control by both predatory invertebrates and forest birds^35–37^. For example, “community-weighted mean body mass” influences ecosystem functioning through the Mass Ratio Hypothesis, which predicts that when ecosystems become degraded, the CWM body mass of avian communities decreases because large-bodied species are filtered first^5, 38^.

**Table 1:** Definition of diversity parameters used on this study^27, 31, 32^.

Parameter	Acronym	Definition	Density weighted
Species richness	*S*	Number of species present in the community	No
Functional richness	FRic	Amount of functional niche volume filled by species in the community	No
Functional evenness	FEve	The evenness of density distribution in filled functional niche volume	Yes
Functional divergence	FDiv	Degree to which density distribution in functional niche volume maximizes divergence in functional traits	Yes
Community-weighted mean	CWM	Average of trait values in the community, weighted by the density of the species carrying each value (see Table 3 for the trait values tested)	Yes

Here, we examine how the taxonomic and functional diversity of avian cavity nesters respond to two types of forest logging in temperate North and South America. We predicted that (a) logging treatments (partial logging, clearcut with reserves) will act as anthropogenic habitat filters of cavity-nesting communities, but (b) the retention of critical stand-level habitat structures by these two treatments will determine the resilience of functional diversity parameters to logging. We finally provide general recommendations to maintain the resilience of tree cavity-using birds and their ecosystem functions in temperate forests of the Americas.

## Materials and Methods

### Study areas

**Figure 2:**
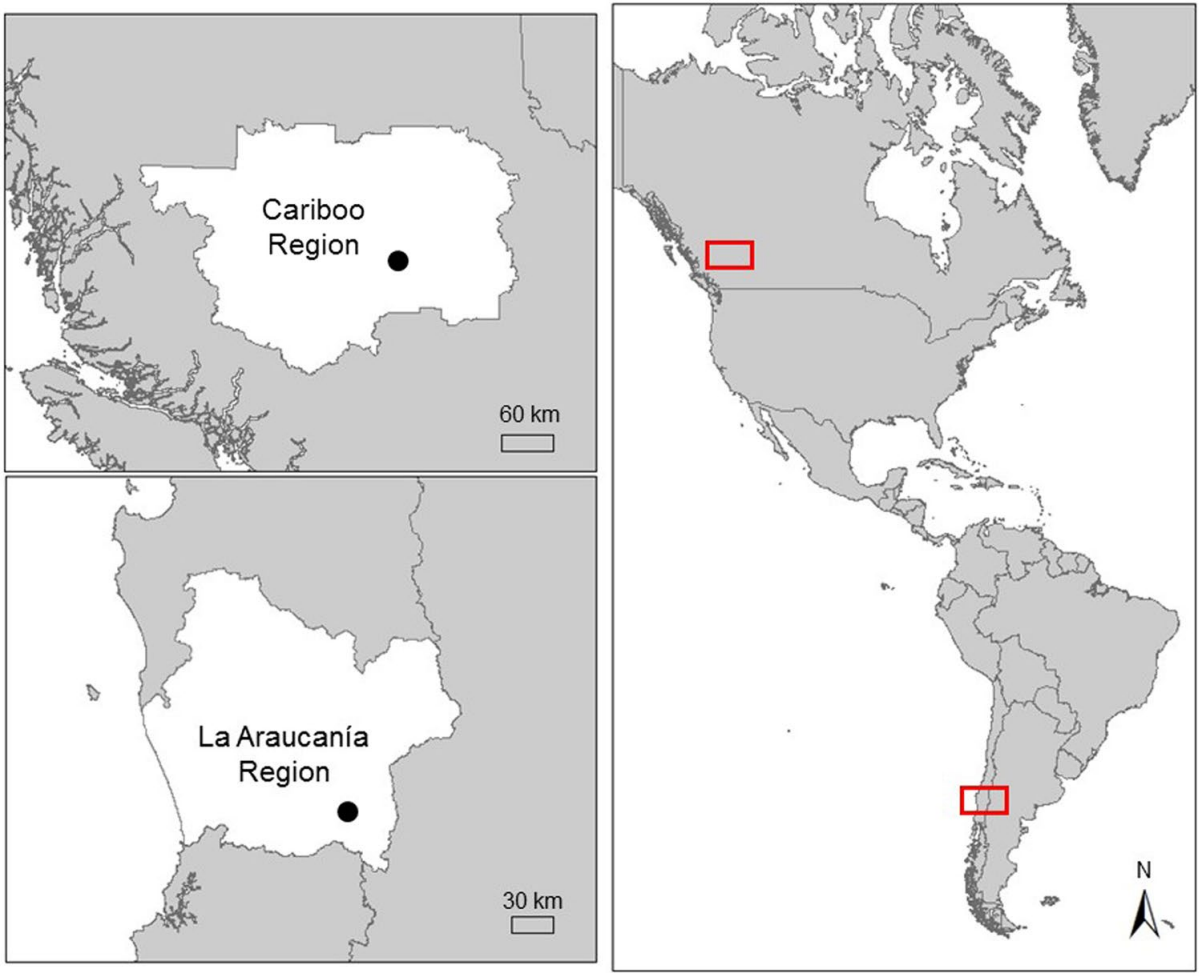
Location of study areas (dots) in the Cariboo Region, British Columbia (BC), Canada (52°08′N 122°08′W), and the La Araucanía Region, Chile (39°16′S 71°48′W), in temperate forests of the Americas. This figure was produced using ArcGIS 10.4.1 (http://support.esri.com/Products/Desktop/arcgis-desktop/arcmap/10-4-1).

Cavity-nesters from North and South American temperate forests have independent evolutionary histories, but they use stands that are comparable in vertical structure and availability of feeding resources^13, 39, 40^. We studied avian cavity-nesters in temperate mixed deciduous/coniferous forests of the Cariboo Region, British Columbia (BC), Canada (52°08′N 122°08′W; 1997–2011), and the La Araucanía Region, Chile^5, 22^ (39°16′S 71°48′W; 2008–2013; Fig. 2; Table 2). Sites in Canada support slightly higher species richness of tree cavity-using birds (32 species)^22^ than the sites in Chile (29 species)^41, 42^. Forests in Canada were dominated by Douglas-fir (*Pseudotsuga menziesii*), lodgepole pine (*Pinus contorta* var. *latifolia*), and hybrid white-Engelmann spruce (*Picea glauca* x *engelmannii*). Deciduous species in Canada included trembling aspen (*Populus tremuloides*), with various alder (*Alnus* spp.), paper birch (*Betula papyrifera*), and willow (*Salix* spp.)^43^. In Chile, low-elevation (<700 m asl) forests were dominated by Dombey’s beech (*Nothofagus bombeyi*), roble beech (*Lophozonia obliqua*), lingue tree (*Persea lingue*), Chilean hazel (*Gevuina avellana*), and Chilean Laurel (*Laurelia sempervirens*). Sites at high elevations (>700 masl) were dominated by either Chilean tepa (*Laureliopsis philippiana*) and Prince Albert’s yew (*Saxegothaea conspicua*) or by monkey puzzle tree (*Araucaria araucana*) and lenga beech (*Nothofagus pumilio*)^5^.

**Table 2:** Stand-level attributes (mean ± SD) that provide habitat for cavity nesters across three logging treatments in temperate forests of (a) Canada and (b) Chile (N = 599 vegetation plots for Canada. N = 355 vegetation plots for Chile).

Country	Stand-level attribute	Logging treatment		
		Uncut	Partial logging	Clearcut
a. CANADA	Tree density (#/ha)	606.9 ± 290.6	471.9 ± 312.7	212.2 ± 257.6
	Diameter at breast height (DBH, cm)	22.2 ± 9.9	21.4 ± 7.7	23.3 ± 12.1
b. CHILE	Tree density (#/ha)	506.8 ± 261.6	423.4 ± 192.0	154.6 ± 145.4
	Diameter at breast height (DBH, cm)	43.4 ± 15.4	31.5 ± 7.5	21.1 ± 10.2

We studied taxonomic and functional diversity on sites under the following treatments: “uncut” (>100 years old), “partial logging” (15–30% tree removal), and “clearcut with reserves” (50–90% tree removal)^19^. In Canada, 27 sites were initially uncut. Between 1997 and 2006, fourteen of these sites were logged (five partially-logged and nine clearcut with reserves; 13 remained uncut; Table 2). British Columbia forest policies stipulate that a portion of the forest stand is retained (left unlogged), either as reserves or as single or small clumps of trees spread throughout the cutblocks. These remnants are comprised mostly of trembling aspen and Douglas*-*fir. In particular, trembling aspen has no commercial value and is considered “critical habitat” for cavity nesters because of the high number of good quality cavities it provides^19, 22^.

In Chile, 26 sites were initially uncut. Between 2002 and 2010, 19 of these sites were logged (10 partially-logged and nine clearcut with reserves; seven remained uncut; Table 2). For Chile, we also added 26 extra sites to the analysis (eight partially-logged and 18 clearcut with reserves. Logging occurred on these additional sites between 1985–1995 and birds were surveyed only after logging. In Chile, forest policies do not consider cavity nesters and stipulate only minimum diameter cutting limits, allowing foresters to remove any trees >10 cm in diameter; thus, large trees were cut and smaller trees (≤10 cm) were retained on site^44^.

### Avian surveys, species trait data, and habitat surveys

We surveyed diurnal cavity-nesting birds using 50-m fixed-radius point-counts. There were 10–32 point-count stations per site in Canada, and 5–10 stations per site in Chile. One or two trained observers conducted one 6-min point-count at each station, each breeding season from 1997 to 2011 in Canada (May to July) and from 2008 to 2013 in Chile (November to February)^5, 19^. One of us (KM in Canada, JTI in Chile) was involved in training all observers over all years in each system (JTI worked in both systems). The same observers surveyed each site to ensure that individual bird counts were consistent among sites. We did not survey during inclement weather (e.g., rain, winds > 20 km hr−1). We validated our sampling effort using sample-based rarefaction accumulation curves (J. T. Ibarra Unpublished Data). We considered our effort to be adequate for measuring species richness in either system when there was no longer an increase in species as individuals accumulate^45^. The accumulation curves were obtained from 1000 random “runs” without replacement, conducted in the program EstimateS^46^. Because the detection rates of birds may be affected by temporal, abiotic, and biotic factors, we suggest that future studies should account for sources of variation in species detectability^47, 48^.

We chose functional traits associated with resource utilization by cavity-nesters, all of which are relevant for ecological interactions and ecosystem functioning (Table 2)^5, 21, 22, 49, 50^. These included categorical (nesting guild, foraging guild, and foraging substrate) and continuous species traits (clutch size [# eggs/clutch], body mass [g], and nest-tree size [tree diameter at breast height, DBH in cm]; Table 3). Nesting guild and nest-tree size are traits that, to our knowledge, have not been used previously in functional diversity studies. Nesting guild refers to the strategy by which birds acquire cavities^22^: (i) strong cavity excavators (e.g., woodpeckers) create their own cavities for nesting; (ii) secondary cavity nesters (e.g., swallows, some ducks, raptors, and parakeets) are unable to excavate their own holes, relying on cavities created by either excavators or tree decay processes; (iii) weak cavity excavators (e.g., nuthatches and some chickadees in Canada, and treerunners in Chile), create their own cavities in soft wood, enlarge one initiated by a stronger excavator or sometimes reuse existing cavities. Tree DBH was used because it is usually considered to be a reliable indicator characteristic related to the production of suitable tree cavities for cavity nesters, the critical ecosystem process needed to sustain diverse cavity-using vertebrate communities^21, 22^.

**Table 3:** Trait values used to measure functional diversity parameters for avian cavity-nesting species from temperate forests of (a) Canada and (b) Chile*.

English name	Scientific name	Nesting guild^a^	Foraging guild^b^	Foraging substrate^c^	Clutch size (mean # eggs/clutch)	Body mass (mean g)	Nest-tree size (mean diameter at breast height DBH, cm)
**a**. **CANADA**							
American kestrel	*Falco sparverius sparverius*	SCN	I	A	4.3	117	42.3
American three-toed woodpecker	*Picoides dorsalis*	SCE	I	B	3.6	65	24.0
Barrow’s goldeneye	*Bucephala islandica*	SCN	M	W	8.0	950	47.5
Black-backed woodpecker	*Picoides arcticus*	SCE	I	B	3.5	70	29.7
Black-capped chickadee	*Poecile atricapillus*	WCE	I	F	6.4	11	20.0
Boreal chickadee	*Poecile hudsonicus*	WCE	I	F	6.1	10	18.4
Brown creeper	*Certhia americana*	SCN	I	B	4.9	8	33.8
Bufflehead	*Bucephala albeola*	SCN	I	W	8.0	473	36.0
Downy woodpecker	*Picoides pubescens*	SCE	I	B	5.6	27	24.8
European Starling	*Sturnus vulgaris*	SCN	O	G	4.7	78	35.5
Hairy woodpecker	*Picoides villosus*	SCE	I	B	3.9	66	30.3
House wren	*Troglodytes aedon*	SCN	I	G	5.9	11	27.6
Mountain bluebird	*Sialia currucoides*	SCN	I	A	5.1	28	32.8
Mountain chickadee	*Poecile gambeli*	WCE	I	F	6.4	11	25.6
Northern flicker	*Colaptes auratus*	SCE	I	G	7.5	135	36.0
Pileated woodpecker	*Dryocopus pileatus*	SCE	I	B	4.3	290	44.7
Pacific-slope flycatcher	*Empidonax difficilis*	SCN	I	A	3.6	11	54.9
Red-breasted nuthatch	*Sitta canadensis*	WCE	I	B	6.0	10	25.5
Red-naped sapsucker	*Sphyrapicus nuchalis*	SCE	S	B	4.8	50	31.2
Tree swallow	*Tachycineta bicolor*	SCN	I	A	5.0	20	30.4
Violet-green swallow	*Tachycineta thalassina*	SCN	I	A	4.4	14	75.1
**b**. **CHILE**							
American kestrel	*Falco sparverius cinnamonimus*	SCN	O	G	4.0	122.5	147.0
Austral parakeet	*Enicognathus ferrugineus*	SCN	F	F	6.5	200	61.7
Austral thrush	*Turdus falcklandii*	SCN	F	G	3.0	78.75	42.4
Bar-winged cinclodes	*Cinclodes fuscus*	SCN	I	G	2.5	29.5	63.6
Black-throated huet-huet	*Pteroptochos tarnii*	SCN	I	G	2	144.33	104.4
Chilean flicker	*Colaptes pitius*	SCE	I	B	4	125	181.1
Chilean swallow	*Tachycineta meyeni*	SCN	I	A	4	16	71.9
Chucao tapaculo	*Scelorchilus rubecula*	SCN	I	G	2	40.35	72.0
House wren	*Troglodytes aedon*	SCN	I	F	5	10.37	41.9
Magellanic tapaculo	*Scytalopus magellanicus*	SCN	I	G	2	11.67	62.0
Magellanic woodpecker	*Campephilus magellanicus*	SCE	I	B	1.5	260	103.4
Plain-mantled tit-spinetail	*Leptasthenura aegithaloides*	SCN	I	B	3	9.1	54.1
Slender-billed parakeet	*Enicognathus leptorhynchus*	SCN	F	F	4.5	250	140.3
Striped woodpecker	*Veniliornis lignarius*	SCE	I	B	3.5	39.97	45.6
Thorn-tailed rayadito	*Aphrastura spinicauda*	SCN	I	B	5	11.74	58.5
White-throated treerunner	*Pygarrhichas albogularis*	WCE	I	B	3	25.6	35.9

*Species trait values and categories were obtained from^21, 22, 40, 42^, and also from J. T. Ibarra and T. A. Altamirano, unpublished data; K. Martin, unpublished data. ^a^SCE = strong cavity excavator, WCE = weak cavity excavator, SCN = secondary cavity-nester. ^b^I = insectivore, F = frugivore, M = molluscivore, O = omnivore, S = sap feeder. ^c^A = air, B = bark, W = water, F = foliage, G = ground.

To measure changes in stand-level attributes across forest treatments, we surveyed trees within plots (11.2 m radius; 0.04 ha) centered at each point-count station. Within each plot we measured the DBH of all trees with a DBH ≥ 12.5 cm. Plots with a radius of 11.2 m are widely used for local ground-based inventory purposes in temperate forests. The rationale behind this plot size is that it is large enough to cover the variability within many temperate forest types, but not so large that it contains so many trees that measurement of those trees is prohibitively expensive^43^. Some jurisdictions have adopted this size as a “standard” for assessing timber volume when fixed-radius plots are used.

### Statistical analysis

To simultaneously interpret results for two systems with different regional species pools and richness, we obtained a random bootstrap sample of 10 point counts (allowing for replacement) per site and year for both Canada and Chile. Counts of individual birds from these 10 sample point-counts were then averaged to derive one species richness (*S*) estimate and one density estimate per site per year. After standardizing each trait measure to a mean of 0 and a standard deviation of 1, we used the program R-FD to estimate functional diversity parameters (FRic, FEve, FDiv, CWM; Table 1) for each forestry treatment^51^. We used R^52^ to compare species richness and functional diversity parameters across treatments, performing one-way repeated measures ANOVA with forestry treatment (uncut, partial logging, and clearcut) as the factor, complemented with Tukey-HSD posthoc tests.

## Results

In temperate forests of Canada and Chile, logging was associated with changes in stand-level attributes that provide habitat for avian cavity nesters (Table 2). With increasing intensity of logging treatments, tree densities declined in both Canada and Chile, but mean DBH declined only in Chile (Table 2).

### Cavity nester response to logging in Canada

**Figure 3:**
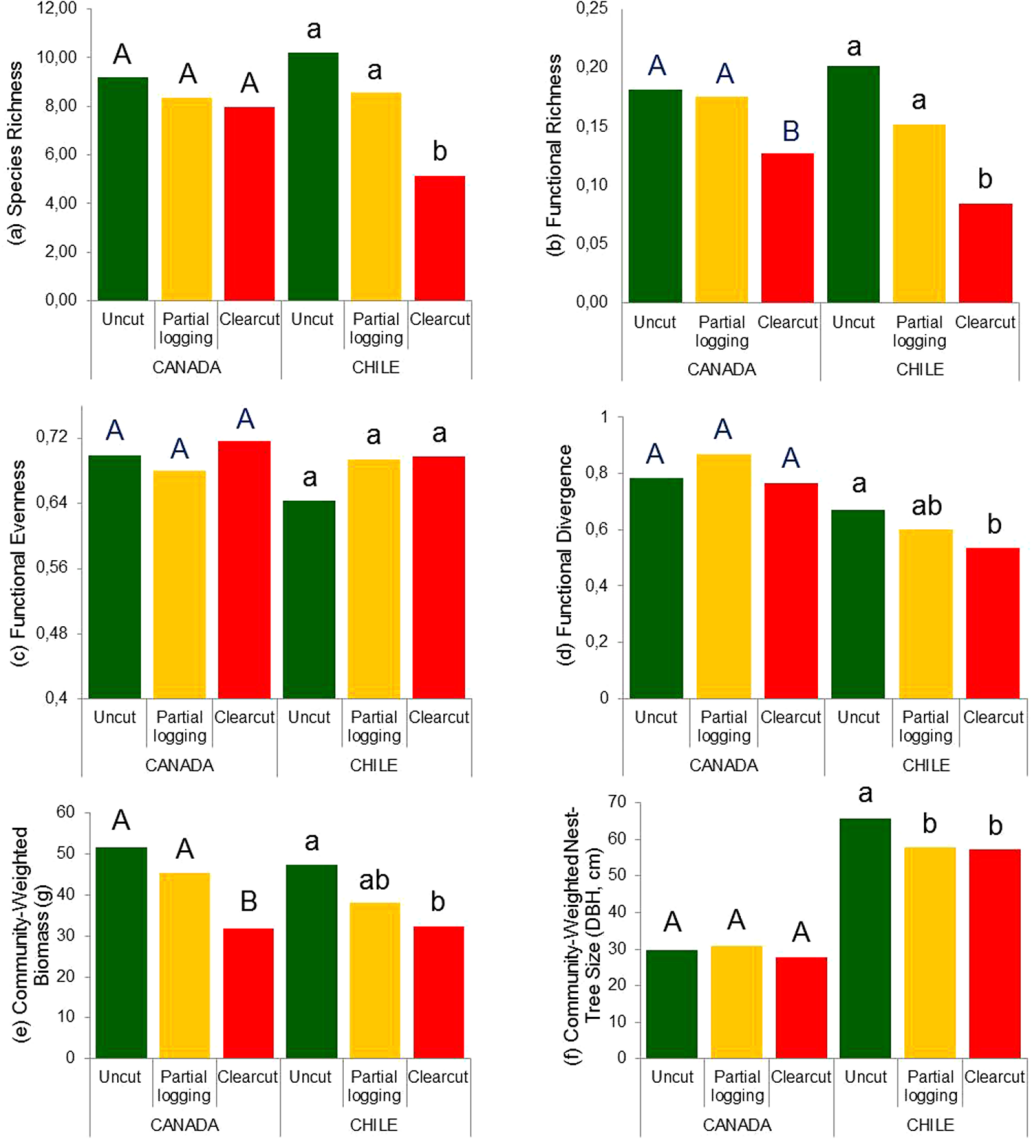
Response of (**a**) species richness [*S*] and five functional diversity indices (**b**) Functional richness [FRic], (**c**) functional evenness [FEve], (**d**) functional divergence [FDiv], (**e**) community-weighted mean [CWM] body mass [g], and (**f**) CWM nest-tree size [diameter at breast height, DBH in cm] to three forestry treatments: “uncut” (green bars), “partial logging” (yellow bars), and “clearcut with reserves” (red bars) in temperate forests of Canada and Chile. Bars with distinct letters (uppercase for Canada; lowercase for Chile) were significantly different according to Tukey-HSD posthoc tests (p < 0.05).

Cavity nester species richness (*S*) did not vary across logging treatments in Canada (uncut mean ± SE: 9.19 ± 2.94 species per site, partially-logged: 8.36 ± 2.0, clearcut: 7.98 ± 1.92; Fig. 3a). Functional richness (FRic) did not differ between uncut and partially-logged sites, but decreased significantly in clearcut sites (uncut: 0.18 ± 0.07, partially-logged: 0.18 ± 0.08, clearcut: 0.12 ± 0.07; F = 10.6, n = 41, p < 0.01; Fig. 3b). Functional evenness (FEve) did not vary across the three treatments (Fig. 3c). Functional divergence (FDiv) showed no differences among logging treatments in Canada (Fig. 3d). Community-weighted mean (CWM) body mass was lower in clearcut sites (31.87 ± 18.02 g) than in partially-logged sites (45.28 ± 16.57 g) and uncut sites (51.72 ± 12.64 g; F = 5.43; n = 41, p < 0.01; Fig. 3e). CWM nest-tree size did not vary across logging treatments in Canada (Fig. 3f).

### Cavity nester response to logging in Chile

Cavity nester species richness (*S*) decreased significantly in clearcut sites in Chile (uncut: 10.19 ± 2.64 species per site, partially-logged: 8.56 ± 1.92, clearcut: 5.15 ± 1.52; F = 22.44, n = 71, p < 0.01; Fig. 3a). Functional richness (FRic) did not differ between uncut and partially-logged sites, but decreased significantly in clearcut sites (uncut: 0.20 ± 0.10, partially-logged: 0.15 ± 0.1, clearcut: 0.08 ± 0.06; F = 11.74, n = 71, p < 0.01; Fig. 3b). Similar to Canada, functional evenness (FEve) did not vary across the three treatments (Fig. 3c). Functional divergence (FDiv) was lower in clearcut sites (0.53 ± 0.12) than in uncut sites (0.67 ± 0.08; F = 4.43; n = 71, p < 0.05; Fig. 3d), with partially-logged sites having intermediate FDiv (0.60 ± 0.12; Fig. 3d). Community-weighted mean (CWM) body mass was lower in clearcut sites (32.40 ± 13.37 g) than in uncut sites (47.28 ± 16.06 g; F = 6.38, n = 71, p < 0.01), and intermediate in partially-logged sites (38.05 ± 16.64 g; Fig. 3e). CWM nest-tree size was lower in both clearcut (57.14 ± 10.04 cm) and partially-logged sites (57.81 ± 6.62 cm), than in uncut sites (65.65 ± 6.44 cm; F = 8.68, n = 71, p < 0.01; Fig. 3f).

## Discussion

Since 1980, considerable scientific and public attention has been directed toward maintaining taxonomic biodiversity - keeping as many species as possible - in managed forests and protected areas^53^. However, recent studies emphasize that loss of functional diversity (technically the diversity of traits, but a concept taken to represent the diversity of species’ niches or functions) is a greater threat to ecosystem resilience than loss of taxonomic diversity^7, 54^. Our study of cavity-nesting birds showed that logging can reduce functional diversity even when taxonomic diversity is maintained. Moreover, we found that functional diversity was maintained better under logging treatments in Canada, where critical habitat structures are targeted for conservation, than in Chile where such critical habitat structures are removed. Our results highlight the importance of retaining critical nesting substrates to conserve functional diversity and maintain the resilience of cavity-nesting communities.

In our study, functional richness (FRic) declined under clearcutting in Canada, even though species richness (*S*) was maintained, confirming the concern that *S* is not always a reliable surrogate for functional richness^6^. Decreasing values of functional richness indicate progressive habitat filtering of certain functional traits^32^, and thus the lower functional richness observed in clearcut areas in both Chile and Canada indicate that clearcutting results in a decrease in the amount of functional niche volume occupied by cavity nesters compared to uncut and partially-logged sites^55^. For example, clearcut sites are readily colonized by opportunistic bird species, usually omnivore or granivore open cup nesters, as new gaps in the niche volume become available in the recently opened areas; opportunistic species rapidly replace more forest-specialized cavity-nesting species^5, 29^. In contrast to clearcuts, partially-logged sites did not decline in functional richness compared to uncut sites, suggesting that partial logging treatments can conserve comparable levels of ecosystem functioning, including foraging and nesting rates, compared to uncut areas^19, 35^. Functional evenness (FEve) can be considered as the degree to which species densities in the cavity-nesting community are distributed in the functional niche volume to allow efficient use of available resources. Our finding that functional evenness was maintained across logging treatments indicates that the distribution of densities of cavity-nesting bird species in functional niche volume may be relatively resilient to logging^5, 27^.

Functional divergence (FDiv) decreases if abundant species are close to the center of the filled functional niche volume^55^. In Chile, uncut sites showed higher functional divergence values than clearcut sites, indicating proportionally higher abundance of species with extreme functional trait values on uncut sites (e.g., species that utilize the largest and the smallest available trees for nesting and feeding; Table 2b). A likely explanation for this finding is that clearcutting reduces resource availability and structural complexity, limiting niche differentiation^5, 18, 28, 47^.

Body mass is inversely related to species’ metabolic rates and population sizes, both parameters related to ecosystem functioning^56^. On a meta-analysis on the effects of logging on avian diversity across the tropics, Burivalove *et al*.^26^ found that larger species of carnivores, herbivores, frugivores, and insectivores responded more negatively to selective logging than smaller species of omnivores and granivores. Our result that clearcutting reduced community-weighted mean (CWM) body mass supports the idea that logging acts as an anthropogenic habitat filter, excluding the largest cavity-nesting birds. As forest patches decrease in size and sparse trees are removed, large-bodied cavity nesters, usually species with more specific habitat requirements, are likely to be filtered out first^5, 29^. In Canada, for instance, retention of relatively small areas of wildlife trees will profit the smaller cavity nesters^57^. In Chile, increasing intensity of logging filters out the largest excavator, Magellanic woodpecker (*Campephilus magellanicus*), and, as a consequence, likely reduces the abundance of large secondary-cavity nesters that use its cavities such as the rufous-legged owl (*Strix rufipes*) and the Austral parakeet (*Enicognathus ferrugineus*)^5^.

We found that community-weighted mean (CWM) nest-tree size did not differ across logging treatments in Canada, but it declined with clearcutting in Chile. A plausible explanation for these results is the differences in the origin of tree-cavities used by secondary cavity nesters, and the difference in logging regulations between Canada and Chile. In our study system in Canada, secondary cavity-nesters most often use cavities excavated by woodpeckers (77%)^21^, in trembling aspen (95% of all tree cavity nests used, mean DBH 30.5 cm)^22^. Logging operations at our study area in Canada retained most aspen and large Douglas-fir, which reduced tree density but maintained mean DBH even in clearcuts, thus conserving key habitat structures for cavity-using vertebrates^41^. In contrast, in our study system in Chile, most nests of secondary cavity-nesters (75%) are in non-excavated cavities generated by tree decay processes in large-decaying trees (mean DBH 57.3 cm)^42^. High quality cavities generated solely by tree decay processes require many years to form^21^. Large trees are cut first during logging operations in Chile, resulting in a decline in mean DBH with increased logging intensity from uncut through partial cutting to clearcuts. Thus, as logging intensity increases in Chile, species requiring large-diameter nest trees are likely to be filtered out of the system.

### A general framework for maintaining the resilience of cavity nester ecosystem functions

A key implication of our results is that models incorporating information on species traits can be used to predict the functional responses of tree cavity-nesting vertebrate communities in areas where forest is being logged. The retention of key habitat structures for cavity nesters (decaying aspen) in Canada buffered shifts in functional diversity. The retention of aspen, a tree species with low commercial value, in areas with commercial forest harvest operations can result in maintaining or increasing densities of woodpeckers, a guild which has been validated as an indicator of both resilient forests and species richness of other forest birds^19, 43^. In contrast, key habitat structures in Chile (large decaying trees) are commonly not retained, and thus functional diversity is affected more adversely, jeopardizing the resilience of ecosystem functions in southern temperate forests. We suggest that environmental and forestry certification agencies should require forestry companies and land owners to implement thoroughly designed logging schemes that ensure the supply of tree species and sizes preferred by cavity nesting vertebrates (including large-bodied species that require the largest trees and smaller species that can be lost from the community if cavity supply is low)^21^. These habitat structures can be conserved by retaining, either dispersed or aggregated, large and small trees (the latter for a continuous supply of large trees) over forest generations, with the aim of maintaining ecosystem functions provided by cavity nesters.
